# Prognostic and predictive role of liquid biopsy in lung cancer patients

**DOI:** 10.3389/fonc.2023.1275525

**Published:** 2024-01-18

**Authors:** Tuncay Goksel, Su Özgür, Aslı Tetik Vardarlı, Altuğ Koç, Haydar Soydaner Karakuş, Taha Reşid Özdemir, Kadri Murat Erdoğan, Ceyda Aldağ, Ali Veral, Berna Komurcuoglu, Pınar Gursoy, Mehmet Emin Arayici, Asim Leblebici, Türkan Yiğitbaşı, Hülya Ellidokuz, Yasemin Basbinar

**Affiliations:** ^1^ Department of Pulmonary Medicine, Ege University Faculty of Medicine, Izmir, Türkiye; ^2^ EgeSAM-Ege University Translational Pulmonary Research Center, Izmir, Türkiye; ^3^ Department of Medical Biology, Ege University Faculty of Medicine, Izmir, Türkiye; ^4^ Department of Translational Oncology, Institute of Oncology, Dokuz Eylul University, Izmir, Türkiye; ^5^ Department of Medical Genetics, Health Sciences University, Izmir Tepecik Research and Training Hospital, Izmir, Türkiye; ^6^ Department of Biology, Ege University Faculty of Science, Izmir, Türkiye; ^7^ Department of Medical Pathology, Faculty of Medicine, Ege University, Izmir, Türkiye; ^8^ Health Sciences University, Dr. Suat Seren Training and Research Hospital of Chest Diseases and Surgery, Izmir, Türkiye; ^9^ Department of Medical Oncology, Faculty of Medicine, Ege University, Izmir, Türkiye; ^10^ Department of Cancer Epidemiology, Institute of Health Sciences, Dokuz Eylul University, Izmir, Türkiye; ^11^ Department of Translational Oncology, Institute of Health Sciences, Dokuz Eylul University, Izmir, Türkiye; ^12^ Department of Medical Biochemistry, Faculty of Medicine, Istanbul Medipol University, Istanbul, Türkiye; ^13^ Department of Preventive Oncology, Institute of Oncology, Dokuz Eylul University, Izmir, Türkiye

**Keywords:** lung cancer, liquid biopsy, mutation, EGFR, real time PCR

## Abstract

**Introduction:**

Lung cancer (LC) is a leading cause of cancer-related mortality worldwide. Approximately 80% of LC cases are of the non-small cell lung cancer (NSCLC) type, and approximately two-thirds of these cases are diagnosed in advanced stages. Only systemic treatment methods can be applied to patients in the advanced stages when there is no chance of surgical treatment. Identification of mutations that cause LC is of vital importance in determining appropriate treatment methods. New noninvasive methods are needed to repeat and monitor these molecular analyses. In this regard, liquid biopsy (LB) is the most promising method. This study aimed to determine the effectiveness of LB in detecting EGFR executive gene mutations that cause LC.

**Methods:**

One hundred forty-six patients in stages IIIB and IV diagnosed with non-squamous cell non-small cell LC were included. Liquid biopsy was performed as a routine procedure in cases where no mutation was detected in solid tissue or in cases with progression after targeted therapy. Liquid biopsy samples were also obtained for the second time from 10 patients who showed progression under the applied treatment. Mutation analyses were performed using the Cobas^®^ EGFR Test, a real-time PCR test designed to detect mutations in exons 18, 20, and 21 and changes in exon 19 of the EGFR gene.

**Results:**

Mutation positivity in paraffin blocks was 21.9%, whereas it was 32.2% in LB. Solids and LB were compatible in 16 patients. Additionally, while no mutation was found in solid tissue in the evaluation of 27 cases, it was detected in LB. It has been observed that new mutations can be detected not only at the time of diagnosis, but also in LB samples taken during the follow-up period, leading to the determination of targeted therapy.

**Discussion:**

The results showed that “liquid biopsy” is a successful and alternative non-invasive method for detecting cancer-causing executive mutations, given the limitations of conventional biopsies.

## Introduction

Lung cancer is the leading cause of cancer-related deaths, accounting for nearly 18% of all cancer-related deaths. Lung cancer is the most frequent fatal cancer in males and the second most common cancer in females (after breast cancer) (1). Despite the development of new treatments and surgical techniques, the diagnosis of lung cancer is usually in the late stages. Five-year overall survival rate range from 15-20%. In addition, survival rates differ depending on the spread of the cancer (2).

Since the beginning of the 2000s, cancer-specific driver mutations have been identified with the elucidation of molecular pathways thought to be effective in the carcinogenesis of lung cancer and many other cancers. The identification of tumor-specific molecular changes represents a new strategy for the molecular diagnosis and treatment of lung cancer. With this approach, it has been demonstrated that the epidermal growth factor receptor (EGFR) plays an important role in the diagnosis and follow-up of lung cancer, and that mutations in the EGFR gene can be used as biomarkers. EGFR encodes a transmembrane glycoprotein, a member of the protein kinase superfamily. It is a receptor for members of the epidermal growth factor family. EGFR is a cell surface protein that binds to epidermal growth factor and induces receptor dimerization and tyrosine autophosphorylation, leading to cell proliferation. Mutations in this gene have been associated with lung cancer. The pathogenic EGFR gene variations range from gene amplification and polysomy to insertions, deletions, and point mutations. These changes may lead to altered regulation of their respective downstream pathways as they cause overexpression and/or persistent activation, followed by the RAS-RAF-MAP- and PIK3-AKT-mTOR- pathways, which are associated with proliferation, invasion, metastatic spread, and tumor angiogenesis (3).

The advent of molecular characterization has ushered in a new era in the targeted treatment of advanced non-small cell lung cancer (NSCLC) patients with oncogene-driven mutations. Molecular tests conducted during diagnosis have become an integral aspect of routine care, guiding treatment decisions by identifying activating mutations in EGFR and BRAF, along with rearrangements in anaplastic lymphoma kinase (ALK) and ROS1. This advancement has streamlined the determination of targeted treatment options for cases where driver mutations are identified. These treatments not only have significantly prolonged the median survival of stage 4 diagnosed cases, extending it from a few months to over 3 years, but have also become standard in patient care. EGFR TKIs emerge as the primary first-line treatment for individuals diagnosed with advanced NSCLC characterized by activating EGFR mutations. Robust evidence supporting the effectiveness of EGFR TKIs has been historically established through randomized controlled trials, demonstrating their superiority over conventional platinum-based chemotherapy. Notably, Gefitinib, Erlotinib, and Afatinib have exhibited improved survival outcomes compared to standard-of-care platinum-based chemotherapy in the first-line setting, displaying superior response rates and significantly enhanced progression-free survival (PFS) (4–10).

However, despite the initial success, the emergence of molecular resistance remains a potential challenge. The intricacies of resistance arise from cellular adaptations that sustain cancer growth, complicating the treatment process. Therefore, it is crucial to comprehensively understand the current molecular profile of the tumor to effectively address these challenges (11).

The 2023 guide of the National Comprehensive Cancer Network (NCCN) recommends molecular testing for all cases diagnosed with advanced non-small cell lung cancer (NSCLC), regardless of clinical characteristics. This testing includes the identification of EGFR, KRAS, and BRAF mutations, as well as rearrangements in ALK and ROS1, and the determination of positive PDL-1 expression (https://www.nccn.org/guidelines/recently-published-guidelines).

Molecular diagnosis is traditionally performed using solid tumor tissues. Liquid biopsy specimens are used to monitor the molecular characteristics of cancer during treatment, as they offer the opportunity for less invasive and less expensive genotyping in cases where there is a potentially insufficient tumor sample (12). Liquid biopsy, also known as liquid or liquid phase biopsy, is defined as the sampling and analysis of nonsolid biological tissues such as blood (13). The detection of executive mutations by liquid biopsy and their use as a prognostic model have been accepted by the US Food and Drug Administration (FDA) for many cancers (14). Before the treatment decision is made, the genetic structure of each patient can be investigated using tissue and liquid biopsies. Liquid biopsy of circulating tumor DNA (ctDNA) for lung cancer is a less invasive method than conventional tissue biopsy for identifying EGFR gene mutations. This study aimed to determine the effectiveness of liquid biopsy in detecting EGFR, an executive mutation that causes lung cancer.

## Materials and methods

### Study group

In this descriptive, cross-sectional, observational study; 146 patients in good condition (ECOG (Eastern Cooperative Oncology Group) Performance Status: 0-1, https://ecog-acrin.org/resources/ecog-performance-status/) and advanced stage (Stage IIIB, IV) diagnosed with non-squamous cell non-small cell lung cancer in Ege University Faculty of Medicine, Department of Chest Diseases and Medical Oncology Department and Suat Seren Chest Diseases and Surgery Training and Research Hospital were recruited. The detailed demographic and clinical characteristics of the patients included in the study were recorded.

### Collection of clinical specimens

For EGFR mutation analysis of solid tissue, the results of EGFR mutation analysis routinely performed before 146 cases were included in the study. For mutation analyses of solid tissue, no procedure other than the routine procedure was performed, and liquid biopsy was performed as a routine procedure in cases where no mutation was detected in solid tissue or in cases with progression after targeted therapy. Liquid biopsy samples were also obtained for the second time from 10 patients who showed progression under the applied treatment.

### EGFR mutation analyses

#### Plasma sample preparation

The average time between the diagnosis of progression and blood drawn was 2 weeks. Haemolyzed and clotted samples were excluded. Peripheral blood samples collected from the subjects in tubes containing 4 mL EDTA were centrifuged at 2000xg for 10 min at room temperature, and the plasma was transferred to a new centrifuge tube.Genomic DNA was extracted from plasma samples according to the spin colon based kit protocol using the Cobas^®^ cfDNA Sample Preparation Kit. EGFR gene mutation analyses from clinical samples from the subjects were performed by real-time PCR using the Cobas^®^ EGFR mutation test v2 (Roche Molecular Diagnostics, Pleasanton, CA, USA) kit, which is designed to detect 42 EGFR variations with high allele-specificity (15). The investigated mutation targets include following: Exon 18 mutations G719X (Cosm ID: 6239, 6252, 6253), exon 19 deletions (Cosm ID: 6210, 6218, 6220, 6223, 6225, 6254, 6255, 12367, 12369, 12370, 12382-12387, 12403, 12416, 12419, 12422, 12678, 12728, 13550-13552, 13556, 18427, 23571, 26038), exon 20 mutations S768I, T790M ve Ex20Ins (Cosm ID: 6240, 6241, 12377, 12378, 12376, 13428, 13558) and exon 21 mutations (Cosm ID: 6224, 6213, 12429). The investigated mutations confer sensitivity to tyrosine kinase inhibitors, while the T790M mutation creates resistance.

#### Solid sample preparation

FFPET specimens are processed, and genomic DNA is isolated using the cobas^®^ DNA Sample Preparation Kit. A deparaffinized 5-μm section of an FFPET specimen is lysed, and nucleic acids are released and protected from DNases. Genomic DNA is bound to a glass fiber filter, and impurities are removed. The adsorbed nucleic acids are washed and eluted for further analysis.

### PCR amplification

The DNA samples extracted from plasma and solid tissue (> 2 ng/μL) are amplified by PCR and analysed in the same process. The test uses specific primers for targeted mutations in the EGFR gene. Target amplification is carried out using a DNA polymerase and involves denaturation, primer annealing, and extension cycles. Amplification occurs only in the regions of the EGFR gene targeted by the primers.

### Automated real-time mutation detection

Real-time PCR technology is used, with fluorescently labeled probes. During amplification, the probes bind to the target DNA and are cleaved, releasing fluorescence. Different reporter dyes are used for detecting mutations, and fluorescence is measured at characteristic wavelengths.

### Selective amplification

AmpErase enzyme and deoxyuridine triphosphate (dUTP) are used for selective amplification. The enzyme recognizes and destroys DNA strands containing deoxyuridine, which is only present in amplicon DNA. This selective amplification ensures accurate detection of target DNA.

### Statistical evaluation of data

Statistical analyses were performed using IBM SPSS Statistics V25.0 (IBM SPSS Statistics for Windows version 25.0. Armonk, NY: IBM Corp.) package program. Descriptive statistics of continuous data are presented using mean, standard deviation, median, minimum, and maximum values, and categorical variables are presented as frequency and percentage values. Chi-square and Exact tests were used to evaluate relationships between categorical variables. Statistical significance was set at p <0.05.

## Results

Of the patients included in the study, 63 (43.2%) were female and 83 (56.8%) were male. While the mean age of women is 60.3 ± 11.8 years, the mean age of men is 60.2 ± 11.5. Among the patients, 56.8% (83) were active smokers, and 15.8% (23) were ex-smokers. Single metastasis was observed in 15.1% (22) of the patients, while multiple metastases were present in 30.1% (44). When evaluating the distribution of metastases, the highest frequencies were noted in the bone (32.8%), lymph nodes (23.3%), trachea, bronchus & lung (11.2%), brain and nervous system (11.2%), and adrenal (11.2%). Concerning TKI1, 94.6% (35) of the patients were taking Erlotinib, and 5.4% (2) were taking Gefitinib. As for TKI2, 7.1% (1) were on Erlotinib, and 92.9% (13) had used Osimertinib (**Table 1**).

**Table 1:** Characteristics of patients.

Variables	n	%
Sex		
Male	83	56.8
Female	63	43.2
Smoking Status		
Active Smoker	28	19.2
Exsmoker	23	15.8
Nonsmoker	31	21.2
Unknown	64	43.8
Number of Metastasis		
Single metastasis	22	15.1
Multiple metastases	44	30.1
No metastasis	80	54.8
Site of metastasis		
Bone	38	32.8
Lymph	27	23.3
Trachea,Bronchus&Lung	13	11.2
Brain and Nervous System	13	11.2
Adrenal	13	11.2
Liver	4	3.4
Peritoneum	4	3.4
Pleura	4	3.4
Received Treatment TKI1		
Erlotinib	35	94.6
Gefitinib	2	5.4
Received Treatment TKI2		
Erlotinib	1	7.1
Osimertinib	13	92.9

The performance status of all patients was evaluated as 0-1 according to the ECOG Performance Status Scale. The mutations detected in the first solid tissue and liquid biopsies of patients are presented in **Table 2**. Accordingly, no mutations were detected in 68.5% (100) of the patients, 11.0% (16) Ex19Del, 9.6% (14) Ex19Del+T790M, 5.5% (8) L858R, 2.7% (4) L858R+T790M, and 2.7% (4) Ex20ins. No mutations were detected in 78.1% (114), 13.7% (20) Ex19del, 1.4% (2) Ex19del+ L858R, 0.7% (1) Ex19Del+T790M, % 0.7 (1) Ex20ins, and 5.5% (8) L858R.

**Table 2:** Mutations detected in liquid biopsy and solid tissue.

	n	%
Liquid biopsy 1		
**Ex20ins**	4	2.7
**L858R+T790M**	4	2.7
**L858R**	8	5.5
**Ex19Del+T790M**	14	9.6
**Ex19Del**	16	11
**Mutation not detected**	100	68.5
**Total**	146	100
Liquid biopsy 2		
**Ex19del**	2	20
**Ex19del+ T790M**	2	20
**Ex20ins**	1	10
**T790M**	2	20
**Mutation not detected**	3	30
**Total**	10	100
Solid Tissue		
**Ex19del**	20	13.7
**Ex19del+ L858R**	2	1.4
**Ex19Del+T790M**	1	0.7
**Ex20ins EGFR**	1	0.7
**L858R**	8	5.5
**Mutation not detected**	114	78.1
**Total**	146	100

No mutation was found in 84 (73.7%) of 114 (100%) cases in which no mutation was found in solid (tissue) biopsy or in liquid biopsy 1. When the mutation distributions in solid biopsy, 1 of 114 cases in which no mutation was detected in solid biopsy were evaluated: Ex19Del in 11 (9.6%) cases, Ex19Del+T790M in 9 (7.9%) cases, Ex20ins in 3 (2.6%), L858R in 6 (5.3%) cases, and L858R in 1 (0.9%) case +T790M mutation was detected (**Table 3**).

**Table 3:** Evaluation of the agreement of solid biopsy and liquid biopsy1.

			Results of liquid biopsy						
			Ex19Del	Ex19Del + T790M	Ex20ins	L858R	L858R + T790M	Mutation not detected	Total
Results of solid biopsy	**Ex19del**	**n**	**5**	**5**	0	0	0	10	20
		**Row**	**25.0%**	**25.0%**	0.0%	0.0%	0.0%	50.0%	100.0%
		**Column**	**31.3%**	**35.7%**	0.0%	0.0%	0.0%	10.0%	13.7%
	**Ex19del + L858R**	**n**	0	0	0	0	0	2	2
		**Row**	0.0%	0.0%	0.0%	0.0%	0.0%	100.0%	100.0%
		**Column**	0.0%	0.0%	0.0%	0.0%	0.0%	2.0%	1.4%
	**Ex19Del + T790M**	**n**	0	0	0	0	0	1	1
		**Row**	0.0%	0.0%	0.0%	0.0%	0.0%	100.0%	100.0%
		**Column**	0.0%	0.0%	0.0%	0.0%	0.0%	1.0%	0.7%
	**Ex20ins**	**n**	0	0	**1**	0	0	0	1
		**Row**	0.0%	0.0%	**100.0%**	0.0%	0.0%	0.0%	100.0%
		**Column**	0.0%	0.0%	**25.0%**	0.0%	0.0%	0.0%	0.7%
	**L858R**	**n**	0	0	0	**2**	**3**	3	8
		**Row**	0.0%	0.0%	0.0%	**25.0%**	**37.5%**	37.5%	100.0%
		**Column**	0.0%	0.0%	0.0%	**25.0%**	**75.0%**	3.0%	5.5%
	**Mutation not detected**	**n**	11	9	3	6	1	**84**	114
		**Row**	9.6%	7.9%	2.6%	5.3%	0.9%	**73.7%**	100.0%
		**Column**	68.8%	64.3%	75.0%	75.0%	25.0%	**84.0%**	78.1%
**Total**		**n**	16	14	4	8	4	100	146
		**Row**	11.0%	9.6%	2.7%	5.5%	2.7%	68.5%	100.0%
		**Column**	100.0%	100.0%	100.0%	100.0%	100.0%	100.0%	100.0%

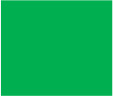
 : Full/Partial Agreement 
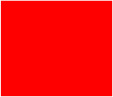
 : Undetected/inconsistent results.

In solid biopsy, Ex19Del mutations were found in 5 (25.0%) of 20 (100%) cases, in which the Ex19del mutation was detected in liquid biopsy and Ex19Del+T790M mutations were detected in 5 (25.0%) cases. In solid biopsy, Ex19del+ L858R mutations detected in 2 (100%) cases were not detected by liquid biopsy 1. Ex19Del+T790M mutations detected in one (100%) case of solid biopsy could not be detected by liquid biopsy 1. In solid biopsy, Ex20ins detected in 1 (100%) were detected as Ex20ins in liquid biopsy 1. In solid biopsy, the L858R mutation was detected in 8 (100%) cases, while in liquid biopsy 1, 2 (25.0%) L858R and 3 (37.5%) L858R+T790M mutations were detected. While full agreement was found in 100 (68.49%) of 146 cases from which solid and liquid biopsy samples were taken, a mutation was found in liquid 1 in 30 (26.32%) of 114 cases in which no mutation was detected in the solid biopsy. In addition, no mutation was found in one liquid biopsy in the 16 cases with mutations in the solid biopsy (**Table 3**).

Ex19Del+T790M mutations were detected in liquid biopsy 2 in one case, whereas no mutation was detected in liquid biopsy 1. Ex19Del was detected in two cases in liquid biopsy 1, while Ex19Del was detected in one of these cases, and Ex19Del+T790M was detected in one case in liquid biopsy 2. Ex19Del was detected in one of the two cases in which the Ex19Del+T790M mutation was detected in liquid biopsy 1, and Ex19Del was detected in two cases of liquid biopsy, and no mutation was detected in one case. While Ex20ins was detected in liquid biopsy 1, the same mutation was found in liquid biopsy 2. While the L858R mutation was detected in one patient in liquid biopsy 1, T790M was detected in liquid biopsy 2 in the same patient. While the L858R+T790M mutation was detected in one case in liquid biopsy 1, the T790M mutation was found in liquid biopsy 2 in the same case. In 99 cases, no mutations were detected in liquid biopsies 1 and 2 (**Table 4**).

**Table 4:** Comparison of mutations detected in liquid biopsy 1 and liquid biopsy 2.

		Liquid Biopsy 2					
		Ex19Del	Ex19Del+T790M	Ex20ins	T790M	Mutation not detected	Total
Liquid Biopsy 1	**Ex19Del n (%)**	1 (50.0)	1 (50.0)	0 (0.0)	0 (0.0)	0 (0.0)	2 (100)
	**Ex19Del+T790M n (%)**	1 (50.0)	0 (0.0)	0 (0.0)	0 (0.0)	1 (50.0)	2 (100)
	**Ex20ins n (%)**	0 (0.0)	0 (0.0)	1 (100)	0 (0.0)	0 (0.0)	1 (100)
	**L858R n (%)**	0 (0.0)	0 (0.0)	0 (0.0)	1 (50.0)	1 (50.0)	2 (100)
	**L858R+T790M n (%)**	0 (0.0)	0 (0.0)	0 (0.0)	1 (100)	0 (0.0)	1 (100)
	**Mutation not detected n (%)**	0 (0.0)	1 (50.0)	0 (0.0)	0 (0.0)	1 (50.0)	2 (100)
**Total n (%)**		2 (20.0)	2 (20.0)	1 (10.0)	2 (20.0)	3 (30.0)	10 (100)

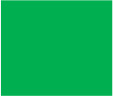
 : Full-Partial Agreement 
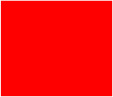
 : Undetected/Inconsistent Results.

When evaluating treatment responses in TKI1 patients, complete response was observed in 20% (7) of those receiving erlotinib, and partial response was noted in 71.4% (25) of cases. In TKI2, progression was observed in one case among patients receiving erlotinib, while partial response was detected in 53.8% (7) of patients receiving Osimertinib. The distribution of survival outcomes according to the presence of mutations is presented in the table. Of the individuals with mutations, 78.7% (37) had deceased, while 70.7% (70) of those without mutations had deceased. Similar survival distributions were observed among the groups based on the presence of mutations (**Table 5**).

**Table 5:** Assessment of patients’ treatment responses and survival outcomes.

Received Treatment * TKI 1 Response		TKI 1 Response				Total	p -value
		Partial	Progression	Stable	Complete		
		n (%)	n (%)	n (%)	n (%)	n (%)	
**Received Treatment** **(TKI 1)**	**Erlotinib**	25 (71.4)	2 (5.7)	1 (2.9)	7 (20.0)	35 (100)	NA
	**Gefitinib**	1 (50.0)	0 (0.0)	0 (0.0)	1 (50.0)	2 (100)	
**Total**		26 (70.3)	2 (5.4)	1 (2.7)	8 (21.6)	37 (100)	
Received Treatment * TKI 2 Response		**TKI 2 Response**				**Total**	**p -value**
		**Partial**	**Progression**	**Stable**	**Complete**		
		**n (%)**	**n (%)**	**n (%)**	**n (%)**	**n (%)**	
**Received Treatment** **(TKI 2)**	**Erlotinib**	0 (0.0)	1 (100)	0 (0.0)	0 (0.0)	1 (100)	NA
	**Osimertinib**	7 (53.8)	3 (23.1)	2 (15.4)	1 (7.7)	13 (100)	
**Total**		7 (50.0)	4 (28.6)	2 (14.3)	1 (7.1)	14 (100)	
Survival		Last status		Total	p -value		
		Ex	Alive	n (%)			
		n (%)	n (%)				
**Mutation status in Liquid Biopsy 1 or 2**	**Mutation (-)**	70 (70.7)	29 (29.3)	99 (100)	0.326		
	**Mutation (+)**	37 (78.7)	10 (21.3)	47 (100)			
**Total**		107 (73.3)	39 (26.7)	146 (100)			

## Discussion

Many studies have investigated the usefulness of liquid biopsy for the molecular diagnosis, monitoring, and determination of targeted therapy strategies in patients with lung cancer. Although recent studies have reported that compatible results were obtained in liquid and tissue biopsies for most patients, the clinical significance of compatible results remains unclear.

Kuo et al. evaluated the predictive factors and clinical outcomes associated with compatible results in liquid/tissue biopsy in newly diagnosed lung adenocarcinoma patients with EGFR gene mutations. While EGFR mutations were detected in 51 tissue samples from 80 patients with stage III or IV lung adenocarcinoma, they showed concordant results in 33 (65.0%) of the liquid biopsy samples taken from these cases (16).

Lin et al. retrospectively analyzed the sequencing results of 100 patients with lung adenocarcinoma (2 cases stage II, 15 cases stage III, and 83 cases stage IV) to compare the sensitivity, specificity, and accuracy of plasma-based NGS testing with solid tumor-based NGS and reported that while 74 clinically significant mutations (94.8% sensitivity) were detected in solid biopsy samples, 41 mutations (52.6% sensitivity) were detected in liquid biopsy samples (17).

Iwama et al. investigated the feasibility of liquid biopsy in determining the activating EGFR mutations that may develop during EGFR inhibitor (afatinib) treatment in patients with lung adenocarcinoma. Tumor and liquid biopsy samples were collected from 32 patients included in the study before treatment, and liquid biopsy samples were collected during the treatment (4 and 24 weeks) and at the end of the treatment. To identify EGFR gene mutations, digital polymerase chain reaction (dPCR) and next-generation sequencing (NGS) analyses were performed on all clinical samples taken from the patients. As a result of NGS analysis, a total of 45 somatic mutations in tumor DNA and 30 somatic mutations in ctDNA were detected; it is also reported that the number of EGFR mutant alleles detected by NGS during treatment is consistent with the allele frequency determined by dPCR. Consistent with the findings of a previous study, the detection sensitivity of somatic mutations from ctDNA to tumor DNA was reported to be 66.7% in this study (18).

Similarly, other studies showed 72.7% and 88% agreement between solid biopsy and liquid biopsy samples, respectively, in detecting EGFR mutations in ctDNA using qPCR in NSCLC patients (19, 20). In this study, we found that the mutation profiles of 100 of 146 cases showed full agreement. In addition, when we compared the results obtained from solid biopsy and liquid biopsy samples taken from the cases at the time of diagnosis in terms of detecting somatic mutations; EGFR gene mutations were detected in 32 (21.9%) solid biopsy samples of 146 cases included in the study, we detected EGFR mutations in 47 (32.2%) liquid biopsy samples.

Next-Generation DNA sequencing technologies have become routinely utilized in the identification of genetic variations, owing to their high accuracy, speed, and extensive sequencing capacity. Particularly in diseases demonstrating genetic heterogeneity, current methodologies allow for a comprehensive approach through a panel that encompasses all genes associated with cancer, revealing the molecular profile of the tumor with a single test. In this study, the utilization of RT-PCR-based analysis instead of the NGS method which NGS is a technology that allows the sequencing of numerous genomic regions, even in samples with low DNA content, in a single test, and consequently, the exclusive examination of EGFR mutations, is perceived as the most significant limitation (21, 22).

Metastatic distributions in NSCLC, particularly in cases of advanced adenocarcinoma of the lung, have been reported to be influenced by various factors, including tumor histology and oncogene status. Some studies have suggested a potential relationship between metastatic distribution and oncogene status, proposing that the biology of NSCLC may guide metastasis among oncogene-dependent cases (23, 24). For instance, patients with EGFR mutations may exhibit distinct metastatic behaviors compared to wild-type tumors: a higher incidence of liver involvement at diagnosis, an increased occurrence of brain metastases initially and/or during the course of the disease, or a tendency to develop widespread/miliary pulmonary metastases (25). In a study conducted by Ochiai and colleagues, the mutational status of EGFR was proposed to influence the recurrence pattern in locally advanced NSCLC post-definitive chemoradiotherapy, with a higher prevalence of distant recurrence among tumors with EGFR mutations compared to EGFR wild-type tumors, which experienced higher rates of local-regional failures (26). Russo and colleagues, in a cohort of 137 cases diagnosed with non-squamous NSCLC, compared different metastatic patterns during the initial phase and progression of the disease based on EGFR mutation status. They reported unique metastatic distributions among EGFR mutation-bearing and EGFR wild-type non-squamous NSCLC, with survival differences according to distinct metastatic behaviors (24). In our study, comparing mutation presence in liquid biopsy according to the number of metastases (single vs. two or more), mutations were detected in 4 cases with a single metastasis, whereas EGFR mutations were identified in the EGFR gene in 20 cases with two or more metastases in patients with stage IV.

Metastasis development did not detected in patients with stage III. These data suggest that tumor biomolecular characteristics and genotypes may impact the metastatic process in NSCLC and, particularly in the presence of limited tissue availability, may contribute to the development of enrichment strategies for tumor genotyping in these tumors.

Re-biopsy is important to investigate resistance mechanisms, especially in NSCLC patients who develop resistance to EGFR-tyrosine kinase inhibitors (TKIs). Susceptible EGFR mutations are among the predictive biomarkers of NSCLC. Therefore, most research and clinical studies conducted to date have focused on EGFR mutations. Studies have reported that deletions in exon 19 and the L858R mutation in exon 21, which are most frequently detected in NSCLC, are associated with sensitivity to small-molecule TKIs, such as erlotinib, gefitinib, and afatinib, as they result in the activation of the tyrosine kinase domain. It has also been shown to be susceptible to EGFR TKI treatment owing to its less common changes, such as exon 19 insertions and point mutations in exon 21 (L861Q, S768I) and exon 18 (G719X). In addition, it has been revealed that cases with EGFR triplet R670W/H835L/L833V mutations, which are rarely detected with the development of new-generation sequencing technologies, can also benefit from TKI. However, some cell clones with EGFR mutations, including most exon 20 insertions, do not respond to EGFR-TKI therapy, and the incidence of these cases constitutes a predictive factor for resistance to the clinically achievable efficacy of TKIs. Moreover, primary resistance to TKI therapy is associated with ALK and ROS1 rearrangements, and KRAS mutations. In addition, approximately 50% of NSCLC patients with EGFR mutations treated with EGFR-TKIs develop acquired resistance to the T790M mutation. It has been reported that acquired resistance may also be associated with histological conversion from NSCLC to small-cell lung cancer (27). The FDA has approved the use of liquid biopsies for the analysis of both susceptible and resistant mutations, and the results strongly support this, in which liquid biopsies are used to guide treatment decisions.

Estimation of treatment outcomes requires the assessment of the levels of both susceptible and resistant mutations. Recent studies have shown that in addition to the detection of T790M mutations, an increase in susceptible EGFR mutations is associated with the diagnosis of progressive disease (19, 28, 29). In addition, another study reported that the incidence of progression was almost five times lower in patients without increased susceptibility to EGFR mutations in plasma, and the risk of death or progression increased almost threefold if the plasma T790M allele frequency increased or occurred, and the increase in susceptibility mutations was associated with T790M resistance, suggesting that it may occur before the detection of the mutation (30).

In a study by Mayo-de-Las-Casas et al., it was reported that among 105 patients who developed resistance to EGFR-TKIs, susceptible mutations and plasma T790M resistance mutations were detected in 56.2% and 35.2% of patients, respectively (31). Timing is important in cancer treatment. In a study using liquid biopsies, progression could be predicted eight months before objective progression when the concentration of the EGFR mutation was observed to increase by ≥20% from the lowest recorded during treatment (32). In addition, early progression can be detected earlier than that detected on computed tomography (CT) scans, as demonstrated by the T790M mutation in plasma.

In a study involving 41 patients, progression was reported in plasma samples 51 days earlier than in computed tomography scans (30). Another study that enrolled 102 patients reported an earlier detection time of 103 days (33).

Ho et al. aimed to evaluate the applicability of quantitative assessment of EGFR driver mutations in plasma in NSCLC patients with EGFR mutations treated with EGFR-TKIs as a tool to evaluate the therapeutic response to TKIs and monitor disease progression; of 136 cases with susceptible EGFR mutation-positive lung adenocarcinoma confirmed by tissue biopsy; Blood samples were taken before TKI treatment and during at least two TKI treatments/follow-up. Plasma samples were analyzed using the cobas^®^ EGFR Mutation Test v2 (cobas^®^ EGFR Test), and semi-quantitative index values for each identified mutation were reported using assay software. The most common basal EGFR mutations detected in the tissue were L858R (53.7%) and exon 19 deletions (39.7%). EGFR mutations were detected in 74% of the initial samples in plasma ctDNA analysis. In this study, an objective response rate with RECIST 1.1 was obtained in 72% of the patients, while the molecular response was reported in 93% of the patients. It was reported that 83% of the patients had molecular progression and 82% of the clinical responders had clinical progression. On average, molecular progression is reported to occur 42 days before clinical progression, and patients who progress during first-line TKI therapy show molecular progression of the original EGFR-susceptible mutations before a T790M mutation appears in 27% of EGFR plasma-positive patients (15).

In our study, we obtained a second liquid biopsy sample from 10 patients who were included in the study and had progressed to detect clinical progression and new EGFR mutations that emerged with treatment. When we evaluated the distribution of mutations detected in liquid biopsy samples taken from these cases, no mutation was detected in three cases, Ex19del mutations were detected in two cases, Ex20ins in one case, T790M in two cases, and Ex19del+T790M mutations in two cases.

When the EGFR variations detected in liquid biopsy 1 and liquid biopsy 2 samples taken from 10 cases with progression were analyzed in detail in terms of molecular response and molecular progression, Ex19Del+T790M mutations were detected in liquid biopsy 2 in one case in which no mutation was detected in liquid biopsy 1. Ex19Del was detected in two cases in liquid biopsy 1, while Ex19Del was detected in one of these cases, and Ex19Del+T790M was detected in one case in liquid biopsy 2. The Ex19Del mutation was detected in one of two cases in which the Ex19Del+T790M mutation was detected in liquid biopsy 1, and the Ex19Del mutation was detected in one case of liquid biopsy. While Ex20ins was detected in liquid biopsy 1, the same mutation was found in liquid biopsy 2. While the L858R mutation was detected in one patient in liquid biopsy 1, T790M was detected in liquid biopsy 2 in the same patient. While the L858R+T790M mutation was detected in one case in liquid biopsy 1, the T790M mutation was found in liquid biopsy 2 in the same case.

These findings reveal that it is possible to monitor the EGFR mutation load with liquid biopsy and can predict both response and clinical progression in lung cancer patients treated with EGFR-TKIs, in whom EGFR mutations are detected, as well as detect treatment-emergent EGFR mutations.

Despite great advances in cancer management, this disease remains one of the world’s most important health problems. Current major challenges include early diagnosis, correct patient stratification, treatment selection, monitoring of treatment response, detection of minimal residual disease, and risk of recurrence. To address these concerns, liquid biopsy-based tools have shown an ever-increasing potential and are of interest to researchers. Liquid biopsy with developing technologies, as it is a non-invasive method that reflects the tumoral molecular profile well, is a suitable method for screening, classification, determining treatment options, monitoring the response to the selected treatment, and developing new oncological tests for the detection of minimal residual disease after surgery and its application in routine clinical practice.

## Data availability statement

The original contributions presented in the study are included in the article/supplementary material. Further inquiries can be directed to the corresponding author.

## Ethics statement

This study was approved by the Istanbul Medipol University Non-Interventional Clinical Research Ethics Committee (Decision date and no: 16/11/2017/10840098-604.01.01-E.43281). The studies were conducted in accordance with the local legislation and institutional requirements. The participants provided their written informed consent to participate in this study.

## Author contributions

TG: Conceptualization, Data curation, Methodology, Validation, Writing – original draft, Writing – review & editing, Funding acquisition, Supervision. SO: Conceptualization, Data curation, Methodology, Validation, Writing – original draft, Writing – review & editing, Formal analysis. AT: Conceptualization, Supervision, Writing – original draft, Writing – review & editing. AK: Conceptualization, Data curation, Methodology, Writing – review & editing. HK: Conceptualization, Data curation, Writing – original draft, Writing – review & editing. TÖ: Conceptualization, Data curation, Writing – original draft, Writing – review & editing. KE: Conceptualization, Data curation, Writing – original draft, Writing – review & editing. CA: Conceptualization, Data curation, Writing – review & editing. AV: Conceptualization, Data curation, Supervision, Writing – original draft, Writing – review & editing. BK: Conceptualization, Data curation, Writing – review & editing. PG: Conceptualization, Data curation, Writing – review & editing. MA: Conceptualization, Data curation, Writing – review & editing. AL: Conceptualization, Data curation, Writing – review & editing. TY: Conceptualization, Data curation, Supervision, Writing – review & editing. HE: Conceptualization, Data curation, Supervision, Writing – review & editing. YB: Conceptualization, Data curation, Methodology, Supervision, Writing – original draft, Writing – review & editing.
